# Chronic mountain sickness in Chinese Han males who migrated to the Qinghai-Tibetan plateau: application and evaluation of diagnostic criteria for chronic mountain sickness

**DOI:** 10.1186/1471-2458-14-701

**Published:** 2014-07-09

**Authors:** Chunhua Jiang, Jian Chen, Fuyu Liu, Yongjun Luo, Gang Xu, Hai-Ying Shen, Yuqi Gao, Wenxiang Gao

**Affiliations:** 1 Department of Pathophysiology and High Altitude Physiology, College of High Altitude Military Medicine, Third Military Medical University, 30 Gaotanyan Street, Shapingba District, Chongqing 400038, P.R. China; 2 Key Laboratory of High Altitude Medicine, Ministry of Education, Third Military Medical University, 30 Gaotanyan Street, Shapingba District, Chongqing 400038, P.R. China; 3 Key Laboratory of High Altitude Medicine, PLA, 30 Gaotanyan Street, Shapingba District, Chongqing 400038, P.R. China; 4 Department of High Altitude Disease, College of High Altitude Military Medicine, Third Military Medical University, 30 Gaotanyan Street, Shapingba District, Chongqing 400038, P.R. China; 5 Robert Stone Dow Neurobiology Laboratories, Legacy Research Institute, Portland, OR 97232, USA

**Keywords:** Hemoglobin, Altitude, Chronic mountain sickness, Hypoxia, Diagnostic criteria

## Abstract

**Background:**

Chronic mountain sickness (CMS), originally characterized by excess hemoglobin (Hb), is currently diagnosed using score-based diagnostic criteria combined with excessive erythrocytosis and clinical symptoms. However, the current criteria have limited applicability. We applied these criteria to 1,029 Chinese Han males migrated to and have been stayed at the Qinghai-Tibet plateau (3,700–5,000 m) for 2–96 months to investigate the prevalence of CMS and its correlations with Hb concentration, altitude, and the length of residence.

**Methods:**

Subjects were screened for CMS using the latest approved diagnostic criteria combined with excessive erythrocytosis and clinical symptoms. Hb concentrations were measured, and a cut-off point was determined with *k*-means clustering. Predisposing factors were evaluated with binary logistic analysis and curve fitting analysis.

**Results:**

***(1)*** The prevalence of CMS at the Qinghai-Tibetan plateau was 17.8% (183/1029 subjects, with CMS score ≥ 6, and Hb ≥ 210 g/L), which is higher than that previously reported. ***(2)*** While individuals were identified into two Hb clusters with a cut-off point of 200 g/L, in the low-Hb cluster (Hb < 200 g/L), the oxygen saturation remained stable as the Hb increased; in the high-Hb cluster (Hb ≥ 200 g/L), the oxygen saturation decreased as the Hb increased. ***(3)*** Two critical factors associated with CMS development were residence at an altitude of 4,500 m and a 60-month length of residence.

**Conclusions:**

Our presenting scoring system is more sensitive than previous diagnostic criteria and favors early screening and treatment of patients with CMS. Our finding suggests that an adjusted Hb threshold of 200 g/L (instead of 210 g/L) is more adaptable in Han individuals at all altitudes. The weight of Hb level should score ≥ 6 points using the CMS scoring system because of the pathophysiologic role of excessive erythrocytosis in patients with CMS. In addition, our data suggest the importance of early screening of CMS via regular medical examinations within the first 60 months of residence at high altitudes, especially >4500 m.

## Background

Chronic mountain sickness (CMS) was first described as “Monge’s disease” by Carlos Monge in 1928 [1], which is characterized by excessive erythrocytosis with the typical symptoms of polycythemia, hypoxemia, breathlessness, palpitations, sleep disturbances, cyanosis, venous dilatation, headaches, tinnitus, and dizziness [2]. There are approximately 140 million people living at high altitudes (>2,500 m) [2] and exposed to the risk of CMS in the world. In the Qinghai-Tibetan plateau of China where the largest population lives at a high altitude worldwide, CMS has been certified as an occupational disease by the Ministry of Health of People’s Republic of China. Patients with CMS lose their ability to adapt to hypoxia and thus are unable to remain stay or full-time work at high altitudes. Therefore, patients must be precisely diagnosed and properly treated as early as possible once the development of CMS initiated.

However, it has been controversial regarding the definition and diagnostic criteria of CMS during the past decades. One focus of this debate is the diagnostic setting of hemoglobin (Hb) threshold for polycythemia. Polycythemia is essentially an adaptive response to long-term hypoxic exposure, making it difficult to be distinguished from a maladaptive response. So far, different Hb thresholds at high altitudes have been used by medical researchers from different groups and countries [3-8]. To unify the definition and diagnostic criteria of CMS, the *Sixth World Congress on Mountain Medicine and High Altitude Physiology* redefined CMS as excessive erythrocytosis (Hb ≥ 190 g/L in female patients; Hb ≥ 210 g/L in male patients), severe hypoxemia, and, in some cases, moderate to severe pulmonary hypertension [9], and a quantitative CMS diagnostic criterion - the CMS score, based on symptoms and the Hb concentration, was also proposed and approved by the International CMS Consensus Working Group [9].

While the CMS scoring system has been applied in a small number of studies to date [4-8], improvement and refinement for the criteria is still needed. For example, Hb thresholds of 200 g/L [7,8] and 210 g/L [4-6] have both been used in male patients. Also, with respect to the essential diagnostic criteria for CMS, whether a sole excessive Hb level, a sole CMS score of ≥6, or both should be applied remains confused. For instance, in one study, subjects were grouped into those with polycythemia (with an increased Hb level) and those with a CMS score of ≥6 [5]. In another study, subjects were grouped according to CMS scores of ≤4, 5 to 9, or ≥10 [10], which is in contrast to the grouping of ≤5, 6 to 10, 10 to 14, or ≥15 recommended by the Consensus Statement [9]. Furthermore, some researchers believe that most of the CMS symptoms are also commonly shared with cardiac, pulmonary, and other diseases, which lead the scoring system less reflect maladaptation to high altitudes after long-term hypoxic exposure, but instead may reflect lung or cardiac malfunction. Therefore, laboratory indices, such as the Hb level, oxygen saturation, and carbon dioxide retention, have been suggested to be more appropriate for CMS scoring [11].

In the presenting cross sectional study, we investigated the prevalence of CMS in 1,029 Chinese Han individuals who migrated to the Qinghai-Tibetan plateau at an altitude of 3,700 to 5,000 m using the latest approved scoring system. In addition, we analyzed the cut-off point of Hb for CMS diagnosis using a data mining method and further discussed the impacts of altitude and length of residence on the prevalence of CMS.

## Methods

### Ethics statement

The current survey was approved by the Ethics Review Board of the Third Military Medical University, Chongqing, China. Separate written informed consent was obtained from all subjects. No health interventions involving the subjects were performed. All individual data were anonymized prior to analysis.

### Study population

Men are reportedly more prone to CMS than are women [12,13]. To exclude a sex difference, we only recruited men in our study. In total, 1,029 male Han Chinese volunteers were recruited from the Qinghai-Tibetan plateau (elevation: 3,700–5,000 m). The study participants were born and previously lived in low-altitude areas, including the Anhui, Beijing, Gansu, Guizhou, Hebei, Henan, Heilongjian, Hubei, Hunan, Jilin, Jiangsu, Jiangxi, Liaoning, Ningxia, Qinghai, Shandong, Shanxi, Shaanxi, Sichuan, Xinjiang, and Chongqing provinces of China. The following basic information about the subjects was obtained using a questionnaire: age (24.08 ± 5.66 years), height (172.11 ± 4.94 cm), and weight (63.72 ± 7.81 kg). All subjects had resided at a high altitude for 2 months–8 years before testing. Individuals with pulmonary or cardiac diseases were excluded. Acute mountain sickness (AMS) and other disease histories, as well as cigarette and alcohol use, were also recorded from the questionnaire. An individual who has consumed at least 100 cigarettes in life was defined a smoker, and who consumes at least 20 g of pure alcohol per day was defined a drinker.

### Measurement of Hb, oxygen saturation, and heart rate

The Hb concentration was determined by cyanide measurement of blood samples obtained from each subject. Each sample was tested in duplicate. Physical parameters were recorded in the sitting position between 9:00 am and 12:00 am. The finger blood oxygen saturation (sO_2_) was measured in the second left finger using a handheld oximeter (TuffSat; GE Healthcare, London, England). The heart rate (HR) and pulse pressure were determined with an electronic sphygmomanometer (HEM-7112; Omron, Kyoto, Japan).

### CMS diagnosis and score calculation

The CMS diagnosis and score calculation were based on published criteria [9]. Briefly, symptoms of breathlessness/palpitations, sleep disturbances, cyanosis, venous dilatation, paresthesia, headache, and tinnitus were weighted (0–3 points) according to severity. The Hb concentration was weighted (0 or 3 points) with a cut-off of 210 g/L. The points for all symptoms and the Hb concentration were summed to yield the CMS score. The severity of CMS was defined as follows: absent, score of 0 to 5; mild, 6 to 10; moderate, 11 to 14; and severe, ≥15 (the ≥15-point score was based on the Consensus Statement). A subject was considered to have CMS only if the Hb concentration was ≥210 g/L and the CMS score was ≥6. A subject was considered not to have CMS if the Hb concentration was <210 g/L or the CMS score was <6.

### Predisposing factors for CMS

Various predisposing factors for CMS have been previously studied, including altitude, sex, age, ethnicity, length of residence, geographic factors, and cigarette smoking [14-16]. Since only Han males residing at the Qinghai-Tibetan plateau were tested in the current study, thus the influences of sex, ethnicity, and geographic factors were exclude. However, the factor of alcohol drinking was not excluded due to it is commonly performed in Han populations at high altitudes. To explore possible predictive parameters for diagnosis, we selected physiological indices, including the sO_2_, HR, body mass index (BMI), and mean blood pressure, which have been proven to be affected by long-term hypoxic exposure. Because CMS symptoms are somewhat nonspecific, we included in the analysis a history of AMS, arthritis, stomach ulceration, cough, headache, cervical osteoarthritis, and other conditions. We pooled all other disease histories and grouped the subjects for comparison with subjects with no disease history because the sample number was not sufficiently large.

### Statistical analysis

We compared the CMS prevalences (%) with the χ^2^ test and the means of two different groups with the independent *t-*test. Data clusters were determined using the *k*-means clustering method. Pearson and Spearman coefficients were used to test bivariate correlations. Curve regression was also performed. The risk of CMS associated with each characteristic was tested by using a two-sided full model logistic regression analysis. All factors were firstly tested for bivariate correlations. Because the age and length of exposure to a high altitude were strongly correlated (data not shown), we omitted age and selected the length of exposure to a high altitude for the analysis. The independent variables which were entered into the final regression model included: altitude, sO_2_, drink, mean BP, BMI, altitude time, HR, AMS history, smoke, and disease history. A p value of <0.05 was considered to be statistically significant.

## Results

### Prevalence of CMS

Of the 1,029 subjects investigated, 183 (17.8%) were diagnosed with CMS, including 101 (9.8%) mild (CMS score of 6–10), 57 (5.5%) moderate (CMS score of 11–14), and 25 (2.4%) severe cases (CMS score of ≥15). Notably, the Hb concentrations and CMS symptoms sometimes deviated from each other. Among 782 subjects with low Hb concentrations (Hb < 210 g/L), 214 had a CMS score of 6 to 10, 36 had a CMS score of 11 to 14, and 11 had a CMS score of ≥ 15 (Table 1).

**Table 1:** CMS score summary

	**CMS score**			
	**< 6**	**6-10**	**11-14**	**≥ 15**
Prevalences (n = 1029)	585	315	93	36
Percentage	56.85%	30.61%	9.04%	3.50%
Hb Mean ± SD (g/L)	191.00 ± 15.01	199.47 ± 20.22	210.41 ± 22.64	217.81 ± 29.95
Hb range (g/L)	146.0-247.0	153.5-269.0	161.5-282.5	167.5-267.5
Hb (g/L) — no. (%)				
< 210 (n = 782)	521 (66.62)	214 (27.37)	36 (4.60)	11 (1.41)
≥ 210 (n = 247)	64 (25.91)	101 (40.89)	57 (23.08)	25 (10.12)
Breathlessness/Palpitations — no. (%)				
0 (n = 442)	376 (85.07)	55 (12.44)	10 (2.26)	1 (0.23)
1 (n = 458)	196 (42.79)	211 (46.07)	44 (9.61)	7 (1.53)
2 (n = 114)	13 (11.40)	46 (40.35)	37 (32.46)	18 (15.79)
3 (n = 15)	0 (0.00)	3 (20.00)	2 (13.33)	10 (66.67)
Sleep disturbance — no. (%)				
0 (n = 467)	400 (85.65)	56 (11.99)	11 (2.36)	0 (0.00)
1 (n = 361)	150 (41.55)	176 (48.75)	32 (8.86)	3 (0.83)
2 (n = 166)	33 (19.88)	76 (45.78)	39 (23.49)	18 (10.84)
3 (n = 35)	2 (5.71)	7 (20.00)	11 (31.43)	15 (42.86)
Cyanosis — no. (%)				
0 (n = 441)	384 (87.07)	54 (12.24)	2 (0.45)	1 (0.23)
1 (n = 347)	159 (45.82)	162 (46.69)	24 (6.92)	2 (0.58)
2 (n = 160)	31 (19.38)	71 (44.38)	46 (28.75)	12 (7.50)
3 (n = 81)	11 (13.58)	28 (34.57)	21 (25.93)	21 (25.93)
Dilatation of veins — no. (%)				
0 (n = 793)	548 (69.10)	212 (26.73)	28 (3.53)	5 (0.63)
1 (n = 161)	31 (19.25)	84 (52.17)	38 (23.60)	8 (4.97)
2 (n = 58)	5 (8.62)	15 (25.86)	22 (37.93)	16 (27.59)
3 (n = 17)	1 (5.88)	4 (23.53)	5 (29.41)	7 (41.18)
Paresthesia — no. (%)				
0 (n = 518)	446 (86.10)	69 (13.32)	2 (0.39)	1 (0.19)
1 (n = 355)	129 (36.34)	178 (50.14)	44 (12.39)	4 (1.13)
2 (n = 119)	8 (6.72)	58 (48.74)	37 (31.09)	16 (13.45)
3 (n = 37)	2 (5.41)	10 (27.03)	10 (27.03)	15 (40.54)
Headache — no. (%)				
0 (n = 435)	382 (87.82)	50 (11.49)	3 (0.69)	0 (0.00)
1 (n = 431)	190 (44.08)	194 (45.01)	44 (10.21)	3 (0.70)
2 (n = 130)	13 (10.00)	65 (50.00)	34 (26.15)	18 (13.85)
3 (n = 33)	0 (0.00)	6 (18.18)	12 (36.36)	15 (45.45)
Tinnitus — no. (%)				
0 (n = 596)	482 (80.87)	101 (16.95)	12 (2.01)	1 (0.17)
1 (n = 336)	98 (29.17)	175 (52.08)	55 (16.37)	8 (2.38)
2 (n = 77)	5 (6.49)	33 (42.86)	23 (29.87)	16 (20.78)
3 (n = 20)	0 (0.00)	6 (30.00)	3 (15.00)	11 (55.00)

### Correlations among Hb concentration, sO_2_, and symptoms

**Figure 1:**
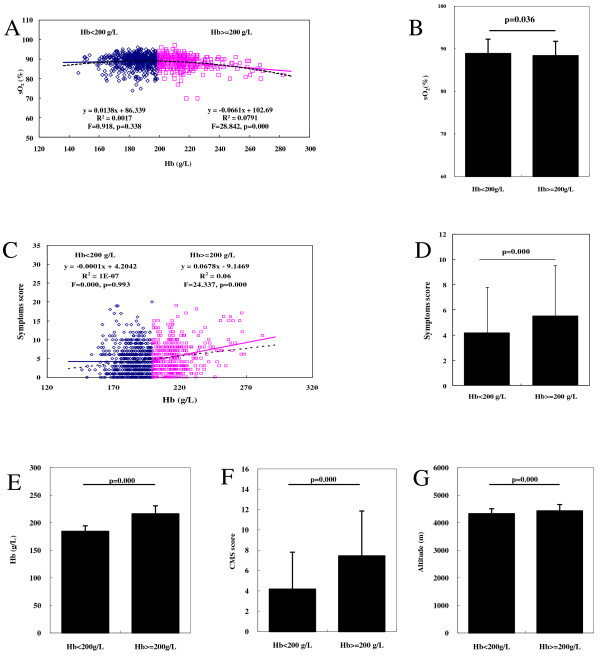
**Correlations among Hb, sO**_**2**_**, and symptoms. (A)** Curve fitting analysis of Hb concentration and sO_2_. **(B)** Mean sO_2_ in the groups with an Hb of <200 and ≥200 g/L. **(C)** Curve fitting analysis of Hb concentration and total symptom score. **(D)** Total symptom score in the groups with an Hb of <200 and ≥200 g/L. **(E)** Hb in the groups with an Hb of <200 and ≥200 g/L. **(F)** CMS scores in the groups with an Hb of <200 and ≥200 g/L. **(G)** Altitude in the groups with an Hb of <200 and ≥200 g/L.

The Hb concentration was significantly correlated with the sO_2_ (Pearson = -0.121, p = 0.000) (Table 2). Curve fitting analysis demonstrated that the best fitting curve was a quadratic curve (F = 16.320, p = 0.000) (Table 2), suggesting a two-phase Hb–sO_2_ relationship according to the Hb value. We then classified the Hb concentration into two clusters using the *k*-means cluster method. The first cluster of Hb was 164.0–202.0 g/L (185.57 ± 10.64 g/L; 95% CI, 200.5–203.4 g/L); the second cluster was 202.3–282.5 g/L (218.68 ± 14.27 g/L; 95% CI, 203.4–251.3 g/L). Based on the above findings, we used 200 g/L as the Hb cut-off point and divided the whole cohort into two clusters: the high Hb cluster (HHbC), comprising subjects with an Hb concentration of ≥200 g/L, and the low Hb cluster (LHbC), comprising subjects with an Hb concentration of <200 g/L. We demonstrated that the Hb concentration, CMS score, and altitude of residence were significantly higher in the HHbC than in the LHbC cluster (p = 0.000, p = 0.000, and p = 0.000, respectively) (Figure 1E–G).

**Table 2:** **Correlation analysis model summary and parameter estimates of Hb with sO**_**2**_

	**R Square**	**F**	**Sig.**	**Constant**	**b1**	**b2**	**b3**
Linear	0.015	13.218	0.000	92.878	-0.021		
Logarithmic	0.012	10.883	0.001	109.176	-3.880		
Inverse	0.010	8.760	0.003	85.163	689.842		
Quadratic	0.035	16.320	0.000	59.677	0.308	-0.001	
Cubic	0.035	16.300	0.000	71.091	0.141	0.000	-0.000
Compound	0.014	12.787	0.000	92.947	1.000		
Power	0.012	10.511	0.001	111.853	-0.044		
S	0.009	8.446	0.004	4.444	7.832		
Growth	0.014	12.787	0.000	4.532	-0.000		
Exponential	0.014	12.787	0.000	92.947	-0.000		
Logistic	0.014	12.787	0.000	0.011	1.002		

Dependent variable: sO_2_; The independent variable is Hb.

Next, we determined the correlations among the Hb, sO_2_, and total symptom score (CMS score minus Hb score) in the two Hb clusters. In the HHbC, sO_2_ was negatively correlated with Hb (Pearson = -0.281, p = 0.000), while the above two parameters were not significantly correlated in the LHbC (Pearson = 0.041, p = 0.338) (Figure 1A); the mean sO_2_ value was significantly higher in the LHbC (88.89 ± 8.37%) than in the HHbC (88.40 ± 3.36%, p = 0.036) (Figure 1B). The total symptom score was significantly correlated with Hb in the HHbC (Spearman = 0.144, p = 0.000) (Figure 1C), but not in the LHbC (Spearman = 0.035, p = 0.993). The total symptom score was significantly lower in the LHbC than in the HHbC (p = 0.000) (Figure 1D).

### Predisposing factors for CMS

We used binary logistic analysis to investigate CMS risk factors, including altitude, length of exposure to a high altitude, cigarette smoking, alcohol drinking, sO_2_, HR, BMI, AMS history, other disease history, and mean blood pressure. The altitude, length of exposure to a high altitude, sO_2_, HR, BMI, alcohol consumption, AMS history, and mean blood pressure were significantly correlated with the prevalence of CMS (p = 0.000, p = 0.021, p = 0.000, p = 0.023, p = 0.001, p = 0.000, p = 0.034, and p = 0.000, respectively). However, cigarette smoking and a history of diseases other than AMS were not significant risk factors for CMS (p = 0.108 and p = 0.909, respectively) (Table 3). Using these factors, we performed binary logistic analysis to derive an anticipation equation (Table 4) with which 93.38% of CMS-vulnerable and 37.60% of nonvulnerable persons can be anticipated (Table 5).

**Table 3:** Binary logistic correlation analysis of risk factors for CMS

	**Score**	**p**
Altitude (meters)	38.5872	0.0000
sO_2_ (%)	25.1352	0.0000
Drink	14.6975	0.0001
Mean BP (mmHg)	13.6726	0.0002
BMI (kg/m^2^)	11.5360	0.0007
Altitude time (months)	5.2909	0.0214
HR (beat/min)	5.1919	0.0227
AMS history	4.4831	0.0342
Smoke	2.5889	0.1076
Disease history	0.0130	0.9092

**Table 4:** Variables in the CMS anticipation equation

	**B**	**S.E.**	**Wald**	**p**	**Exp(B)**	**95.0% C.I. for EXP(B)**		**Cox & Snell R Square**	**Nagelkerke R Square**
						**Lower**	**Upper**		
Altitude	0.0025**	0.0005	20.6871	0.0000	1.0025	1.0014	1.0036	0.1811	0.2602
Altitude time	0.0061*	0.0031	3.8763	0.0490	1.0061	1.0000	1.0123		
Smoke	-0.0600	0.1670	0.1311	0.7172	0.9415	0.6974	1.3053		
Drink	1.0292*	0.2601	15.6731	0.0000	2.7980	1.6811	4.656		
sO_2_	-0.1388**	0.0392	12.5181	0.0004	0.8704	0.8059	0.9399		
HR	0.0019	0.0090	0.0426	0.8364	1.0019	0.9844	1.0196		
BMI	0.0806	0.0561	2.0610	0.1511	1.0839	0.9710	1.2100		
AMS history	-0.5221	0.2714	3.7003	0.0544	0.5933	0.3485	1.0099		
Disease history	-0.3839	0.3877	0.9806	0.3221	0.6812	0.3186	1.4564		
Mean BP	0.0214	0.0123	3.0455	0.0810	1.0216	0.9974	1.0465		
Constant	-4.1097	4.8249	0.7255	0.3943	0.0164				

**Table 5:** Model detection of the CMS anticipation equation (binary logistic analysis)

**Observed**	**Predicted**		**Percentage correct**	**χ**^**2**^	**p**
	**CMS**	**Non-CMS**			
CMS	296.0000	21.0000	93.3754	88.3319	0.000
Non-CMS	78.0000	47.0000	37.6000		
Overall percentage			77.6018		

**Figure 2:**
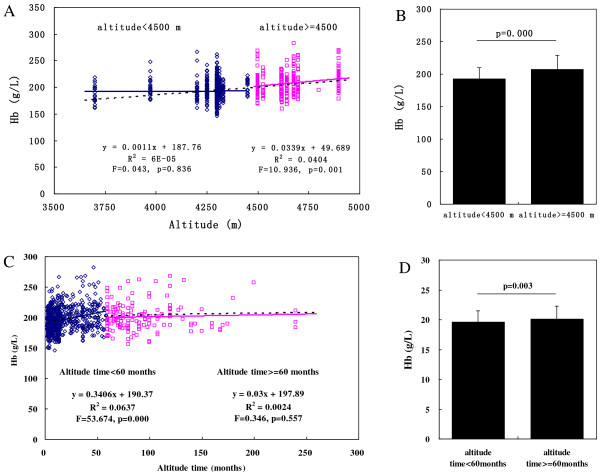
**Correlations of Hb, symptom score, and CMS score with altitude and length of residence at high altitude. (A)** Curve fitting analysis of Hb concentration and altitude. **(B)** Hb concentration in the groups at an altitude of <4500 and ≥4500 m. **(C)** Curve fitting analysis of Hb concentration and length of exposure to high altitude. **(D)** Hb concentration in the groups with a length of exposure to high altitude of <60 and ≥60 months. **(E)** Curve fitting analysis of CMS score and altitude. **(F)** CMS score in the groups at an altitude of <4500 and ≥4500 m. **(G)** Curve fitting analysis of CMS score and length of exposure to high altitude. **(H)** CMS score in the groups with a length of exposure to high altitude of <60 and ≥60 months.

Because height and the duration of exposure to a high altitude were found to be correlated with the CMS score, we proceeded to cluster these two parameters with the *k*-means cluster method and found cut-offs of 4,500 m and 60 months, respectively. In the cluster of an altitude of <4500 m, the Hb concentration was not significantly correlated with the height (F = 0.042, p = 0.836) with a relatively low mean Hb concentration (192.5 ± 1.72 g/L), while it was significantly correlated with height (F = 10.936, p = 0.001) in the cluster of an altitude of ≥4500 m with the mean value exceeded our cut-off point (207.4 ± 21.8 g/L) (Figure 2A,B). In the cluster of an altitude time of <60 months, the Hb concentration was significantly correlated with the length of residence (F = 53.674, p = 0.000) with a low mean value, while it was not significantly correlated with the length of residence (F = 0.346, p = 0.557) in the cluster of an altitude of ≥60 months with the mean value of >200 g/L (Figure 2C,D). Interestingly, there was a negative correlation between the CMS score and altitude in the <4500-m cluster (F = 12.361, p = 0.000) and the mean CMS score remained <6 points. While the CMS score increased according to the altitude in the other cluster (F = 10.816, p = 0.001) with a mean CMS score of about 8 (Figure 2E,F). On the other hand, the CMS score increased rapidly within 60 months after ascent to a high altitude (F = 148.883, p = 0.000) and broke the 6-point threshold after approximately 2 years (y = 0.1137x + 3.0812) (Figure 2G). After 60 months, the mean CMS score exceeded 8 points and increased rather slowly as the exposure time was prolonged (F = 7.446, p = 0.007) (Figure 2G,H).

## Discussion

In past decades, thousands of patients with CMS have been investigated with a focus on the pathophysiologic mechanisms of this medical condition. Nevertheless, a number of questions remain to be elucidated. In the current study, we used the latest diagnostic criteria of CMS to evaluate 1,029 Chinese Han males who had been residing at Qinghai-Tibet plateau (3,700–5,000 m) for 2 months to 8 years. The prevalence of CMS in above population was 17.8% at 3,700 to 5,000 m, which is higher than that of previous report with a prevalence of 11.83% at 4,006 to 5,226 m [17] at the Qinghai-Tibetan plateau. Our results using the latest diagnostic criteria suggest that present scoring system is more sensitive than previous diagnosis criteria, and favors early screening and treatment of patients with CMS. Our data demonstrate that 200 g/L is the optimal for a cut-off point of Hb concentration in Han males residing at a high altitude though a cut-off of Hb concentration ≥210 g/L in male patients has been used as the most important criterion for CMS in the published Consensus Statement [9]. We found a discrepancy between CMS symptoms and an excessive Hb level (≥210 g/L) in some subjects and characterized the effect patterns of height and length of residence at a high altitude on the Hb concentration and CMS prevalence.

### Debate on Hb threshold selection

Several CMS scoring systems have been suggested [18-20] while the 210 g/L has mainly been used as the diagnostic criterion for male patients [9]. Previously reported criteria were most often calculated by the mean Hb concentration plus 2, 3, or 4 times the standard deviation (SD) of the entire population [18,21]. A Chinese group selected an Hb threshold of 190 g/L (mean plus twice the SD) in Tibetans living at a high altitude as a reference for immigrants [22], designating Tibetans as the best high altitude-adapted population [23]. An Hb threshold of 200 g/L (mean plus 3 times the SD) has also been proposed [17]. Although calculation of the threshold with the mean plus a specific multiple of the SD at sea level is a commonly used method, the Hb concentration increases according to the elevated altitude both physically and pathologically. Individuals with an excessively increased Hb concentration could “pollute” the Hb distribution among the whole cohort, resulting in an artificial threshold derived from the mean and SD in addition to the subjective selection of the SD. For example, the Hb concentration was 20.74 ± 2.18 g/dL in men who resided above 4,500 m, and the threshold point was as high as 25.1 g/dL using the mean plus twice the SD. The Tibetan threshold is not a good reference because the Tibetans are resistant to CMS and may not be comparable with the Hans and other high-altitude populations [24].

A data-mining analysis technique was introduced in this study to calculate the Hb threshold based on the “optimal” Hb concentration concept [25]. When a human ascends to a high altitude, adaptive reactions begin to ameliorate hypoxemia owing to the low oxygen partial pressure of inhaled air. Hypoxemia can upregulate the transcription factor hypoxia inducible factor-1 and induce a number of hypoxia-inducible genes, including erythropoietin [26]. The release of erythropoietin leads to erythrocytosis and an increase in the Hb concentration [27] so that the blood is able to carry more oxygen to tissues and cells for utilization. Conversely, excessive erythrocytosis can also increase blood viscosity and slow blood velocity, which may decrease the cardiac output [25] and aggravate arterial hypoxemia, slowing the blood supplication to tissues. This suggests the existence of an “optimal” Hb concentration; i.e., when the Hb concentration is below the “optimal” value, physiologic erythrocytosis response helps to elevate ability of blood to transport oxygen. When the Hb exceeds this optimal value, the erythrocytosis response aggravates the oxygen supplication-demanding imbalance and triggers CMS pathologic processes.

*K*-means clustering is a statistical method used to partition observations into clusters with the nearest means, which serve as prototypes of the clusters. In this study, we were able to completely separate the subjects into two populations (Hb of <200 and ≥200 g/L) using *k*-means clustering analysis; subsequent analyses showed that the 95% CIs were clearly nonoverlapping. In this sense, the nonoverlapping range can serve as a threshold for the different dynamics of the two populations. When the Hb level drifts beyond the 95% CI, further drifting away from the first population will lead to marked changes in the Hb–sO_2_ relationship. In the first population (Hb < 200 g/L), which represents the physiologic response of erythrocytosis, the sO_2_ remains stable as Hb increases; in the second population (Hb ≥ 200 g/L), which represents the pathologic response part of erythrocytosis, the sO_2_ decreases as the Hb increases. The correlations between Hb and the symptoms in the two subpopulations further confirm the marked changes in the Hb–sO_2_ relationship. Still, more remains to be elucidated regarding the Hb threshold, such as that in female individuals and native highlanders, including Tibetans and Andeans.

### Discrepancy between Hb concentration and symptoms

Our data also showed a discrepancy between the Hb concentration and CMS symptoms. Some subjects had a very high Hb concentration but could not be classified as patients with CMS because their CMS symptoms were obscure. Excessive erythrocytosis *per se* represents a threat to humans, sometimes even a lethal threat, because of the risk of development of a thrombus or embolism in vital organs [28,29]. If the diagnosis of CMS cannot be made, dangerous health conditions might be disregarded and proper medical treatment will not be rendered. Some individuals with low Hb concentrations and severe CMS symptoms could not be diagnosed with CMS. Although we found significant correlations between the Hb concentration and each CMS symptom, all were rather weak with the exception of cyanosis (data not shown). Therefore, there may be a low-Hb subtype of CMS that differs from the present subtype characterized by a high Hb concentration; i.e., Monge’s disease or polycythemia. In the high-Hb subtype, excessive erythrocytosis is the primary risk factor; in the low-Hb subtype, other risk factors such as long-term insufficient oxygen supplication to vital organs are likely to be fundamental causes [30].

CMS was first termed “erythema syndrome of high altitude” [1] before being termed CMS in 1998 [31]. CMS was then further divided into two subtypes: CMS and high-altitude pulmonary hypertension [32]. However, this division was confusing because CMS shares the same term as one of its subtypes. It would be clearer to name this subtype of CMS as characterized by excessive Hb polycythemia, the original term.

CMS may manifest as high-altitude pulmonary edema in about 1% of subjects with a low Hb concentration [24], and these subjects could erroneously be classified as having CMS using the scoring system for diagnosis. Moreover, although CMS (polycythemia) is characterized by excessive erythrocytosis, its importance is weakened or “diluted” by multiple symptoms based on the present diagnostic criteria. The weight of three points for excessive polycythemia is lower than the weight of non-specific symptoms, such as sleep disturbances, headache, and dizziness, which can be found in a large number of pathophysiologic conditions. Individuals with excessive polycythemia, even those with very severe polycythemia, may be missed, and the optimal treatment time before the patient shows CMS symptoms may be missed. Based on the above considerations, we suggest that Hb should be given greater weight in the scoring system; for example, 6 points should be given for excessive polycythemia.

### Impacts of height and length of residence at a high altitude on CMS

We used binary logistic analysis to investigate the predisposing factors for CMS. Among these factors, we found that the altitude, length of residence at a high altitude, alcohol use, and sO_2_ were significantly correlated with the prevalence of CMS. We then analyzed the impacts of height and length of residence at a high altitude on CMS.

CMS usually occurs in long-term residents at a high altitude of >3000 m. In one study, as the altitude increased, the prevalence of CMS increased among Han immigrants in the Qinghai-Tibet plateau (2,980–5,226 m) [17]. However, our data followed this pattern only when the altitude was ≥4500 m. The altitude was not associated with the CMS prevalence or erythrocytosis at an altitude of <4500 m. Thus, a significant number of humans can become well acclimatized to hypoxic conditions at <4500 m by maintaining a stable Hb concentration (192.5 ± 17.2 g/L) below the “optimal” value (200 g/L) irrespective of the altitude itself (Figure 2A,B). While the altitude was ≥4500 m and the Hb concentration was mostly ≥200 g/L (207.3 ± 21.8 g/L), excessive erythrocytosis can trigger a vicious circle by impairing oxygen delivery and depressing cellular oxygen supplication. Thus, 4,500 m should be regarded as the threshold altitude for dwelling, above which most humans cannot acclimatize to such a high altitude.

CMS usually requires a specific duration of residence to develop at a high altitude. A Han man can develop CMS in <1 year at 5,300 m (2 months in the current study), while others may not show obvious CMS symptoms until they have lived at a high altitude for ≥15 years (30 years in this study) [17]. Some researchers are convinced that the length of exposure to a high altitude is not associated with the prevalence of CMS, whereas altitude, sex, and ethnic background are significant factors influencing the rate of CMS development [14]. Our data definitely suggest that the length of residence at a high altitude is significantly correlated with CMS development, especially within the first 60 months because the Hb concentration increases rapidly during this time period. As a human being first ascends to high altitude, the body immediately develops hypoxemia, which triggers adaptive responses including hyperventilation, pulmonary hypertension, and an increase in the cardiac output. As time passes and chemosensitivity becomes blunted, the above responses are substituted by erythrocytosis, angiogenesis, and other processes. The erythrocyte and Hb levels continue to increase to a very high point unless the oxygen demand of the body is met. According to our data, this mainly occurs within 60 months of living at a high altitude. Thereafter, the rate of increase in the Hb concentration slows down significantly, and the CMS prevalence gradually stabilizes. Therefore, medical examinations should be regularly performed within the first 60 months of residing at a high altitude. Spending the first 60 months at a high altitude without a CMS incident ensures a much lower probability of the subsequent development of CMS.

## Conclusions

The high prevalence of CMS evaluated in this study using the latest approved scoring system [9] suggests that it is more sensitive than previous diagnosis criteria and favors early screening and treatment of patients with CMS. Based on our data, we suggest that the Hb threshold for CMS is 200 g/L despite the altitude difference and that Hb is weighted with >6 points in the CMS scoring system. Moreover, an elevation of >4500 m and the first 60 months after ascending are two critical factors associated with the development of CMS in long-term residents. Regular medical examinations should be performed within the first 60 months of residing at a high altitude, especially ≥4500 m, for early screening of CMS. To be noticed, our study was focusing on younger subjects (24.08 ± 5.66 years), which may limit the ability to generalize and compare our results to other data sets. More studies with larger population and from different nations are needed to validate the above findings.

## Abbreviations

AMS: Acute mountain sickness; BMI: Body mass index; CMS: Chronic mountain sickness; Hb: Hemoglobin; HR: Heart rate; sO_2_: Oxygen saturation.

## Competing interests

The authors declare that they have no competing interests.

## Authors’ contributions

CHJ designed and performed the research, analyzed the data, and wrote the paper. JC, YQG, and WXG designed the research, analyzed the data, and wrote the paper. FYL, YJL, and GX performed the research. HYS analyzed the data. All authors contributed significantly to the research and review of the final results. All authors read and approved the final manuscript.

## Pre-publication history

The pre-publication history for this paper can be accessed here:

http://www.biomedcentral.com/1471-2458/14/701/prepub

## References

[B1] Monge CC Whittembury J Chronic mountain sickness Johns Hopkins Med J 1976 139 SUPPL 87 89 1011412

[B2] Wu TY Chronic mountain sickness on the Qinghai-Tibetan plateau Chin Med J (Engl) 2005 118 2 161 168 15667803

[B3] Pasha MA Newman JH High-altitude disorders: pulmonary hypertension: pulmonary vascular disease: the global perspective Chest 2010 137 6 Suppl 13S 19S 20522576 10.1378/chest.09-2445

[B4] Leon-Velarde F McCullough RG McCullough RE Reeves J Proposal for scoring severity in chronic mountain sickness (CMS) Adv Exp Med Biol 2003 543 339 354 10.1007/978-1-4419-8997-0_24 14713133

[B5] Groepenhoff H Overbeek MJ Mule M Van der Plas M Argiento P Villafuerte FC Beloka S Faoro V Macarlupu JL Guenard H de Bisschop C Martinot JB Vanderpool R Penaloza D Naeije R Exercise pathophysiology in patients with chronic mountain sickness Chest 2012 142 4 877 884 10.1378/chest.11-2845 22302297

[B6] Kong FY Li Q Liu SX Poor sleep quality predicts decreased cognitive function independently of chronic mountain sickness score in young soldiers with polycythemia stationed in Tibet High Alt Med Biol 2011 12 3 237 242 10.1089/ham.2010.1079 21962067

[B7] Li X Pei T Xu H Tao F You H Liu Y Gao Y Ecological study of community-level factors associated with chronic mountain sickness in the young male Chinese immigrant population in Tibet J Epidemiol 2012 22 2 136 143 10.2188/jea.JE20110058 22343324 PMC3798592

[B8] Pratali L Rimoldi SF Rexhaj E Hutter D Faita F Salmon CS Villena M Sicari R Picano E Allemann Y Scherrer U Sartori C Exercise induces rapid interstitial lung water accumulation in patients with chronic mountain sickness Chest 2012 141 4 953 958 10.1378/chest.11-0084 21885723

[B9] Rimoldi SF Rexhaj E Pratali L Bailey DM Hutter D Faita F Salmon CS Villena M Nicod P Allemann Y Scherrer U Sartori C Systemic vascular dysfunction in patients with chronic mountain sickness Chest 2012 141 1 139 146 10.1378/chest.11-0342 21700688

[B10] Leon-Velarde F Maggiorini M Reeves JT Aldashev A Asmus I Bernardi L Ge RL Hackett P Kobayashi T Moore LG Penaloza D Richalet JP Roach R Wu T Vargas E Zubieta-Castillo G Zubieta-Calleja G Consensus statement on chronic and subacute high altitude diseases High Alt Med Biol 2005 6 2 147 157 10.1089/ham.2005.6.147 16060849

[B11] Gonzales GF Tapia V Gasco M Gonzales-Castaneda C Serum testosterone levels and score of chronic mountain sickness in Peruvian men natives at 4340 m Andrologia 2011 43 3 189 195 10.1111/j.1439-0272.2010.01046.x 21486396

[B12] Zubieta-Castillo G Sr Zubieta-Calleja GR Jr Zubieta-Calleja L Chronic mountain sickness: the reaction of physical disorders to chronic hypoxia J Physiol Pharmacol 2006 57 Suppl 4 431 442 17072074

[B13] Beall CM Goldstein MC Hemoglobin concentration of pastoral nomads permanently resident at 4,850-5,450 meters in Tibet Am J Phys Anthropol 1987 73 4 433 438 10.1002/ajpa.1330730404 3661681

[B14] Wu T Wang X Wei C Cheng H Li Y Ge D Zhao H Young P Li G Wang Z Hemoglobin levels in Qinghai-Tibet: different effects of gender for Tibetans vs Han. J Appl Physiol 2005 98 2 598 604 10.1152/japplphysiol.01034.2002 15258131

[B15] Leon-Velarde F Gamboa A Chuquiza JA Esteba WA Rivera-Chira M Monge CC Hematological parameters in high altitude residents living at 4,355, 4,660, and 5,500 meters above sea level High Alt Med Biol 2000 1 2 97 104 10.1089/15270290050074233 11256567

[B16] Okumiya K Sakamoto R Kimura Y Ishine M Kosaka Y Wada T Wada C Nakatsuka M Ishimoto Y Hirosaki M Kasahara Y Konno A Chen W Fujisawa M Otsuka K Nakashima M Wang H Dai Q Yang A Qiao H Gao J Li Z Zhang Y Ge RL Matsubayashi K Comprehensive geriatric assessment of elderly highlanders in Qinghai, China II: the association of polycythemia with lifestyle-related diseases among the three ethnicities Geriatr Gerontol Int 2009 9 4 342 351 10.1111/j.1447-0594.2009.00555.x 20002753

[B17] Wu T Li W Li Y Ri-Li G Cheng Q Wang S Zhao G Wei L Jin Y Don G Ohno H, Kobayashi T, Masuyama S, Nakashima M Epidemiology of chronic mountain sickness: ten years’ study in Qinghai-Tibet Progress in Mountain Medicine and High Altitude Physiology 1998 Matsumoto, Japan: Press Committee of the 3rd World Congress on Mountan Medicine and High Altitude Physiology 120 125

[B18] Wu T Proposal for CMS Guidilines Discussion, CMS Consensus Working group VI World Congress on Mountain Medicine and High Altitude Physiology 2004 China: Xining, Qinghai

[B19] Leon-Velarde F Ohno H, Kobayashi T, Masuyama S, Nakashima M The diagnositic criteria for CMS Progress in Mountain Medicine and High Altitude Physiology 1998 Matsumoto, Japan: Press Committee of the 3rd World Congress on Mountain Medicine and High Altitude Physiology 160

[B20] Aldashev A Kyrgyzian Proposal for CMS Guidelines Discussion, CMS Consensus Working Group VI World Congress on Mountain Medicine and High Altitude Physiology 2004 China: Xining, Qinghai

[B21] Monge C Leon-Velarde F Arregui A Increasing prevalence of excessive erythrocytosis with age among healthy high-altitude miners N Engl J Med 1989 321 18 1271 2797095 10.1056/NEJM198911023211819

[B22] Wu T Li W Wei L Ri-Li G Wang S Cheng Q Jin Y Ohno H, Kobayashi T, Masuyama S, Nakashima M A preliminary study on the diagnosis of chronic mountain sickness in Tibetan populations Progress in Mountain Medicine and High Altitude Physiology 1998 Matsumoto, Japan: Press Committee of the 3rd World Congress on Mountan Medicine and High Altitude Physiology 337 342

[B23] Beall CM Andean, Tibetan, and Ethiopian patterns of adaptation to high-altitude hypoxia Integr Comp Biol 2006 46 1 18 24 10.1093/icb/icj004 21672719

[B24] Naeije R Vanderpool R Pulmonary hypertension and chronic mountain sickness High Alt Med Biol 2013 14 2 117 125 10.1089/ham.2012.1124 23795731

[B25] Lenfant C Sullivan K Adaptation to high altitude N Engl J Med 1971 284 23 1298 1309 10.1056/NEJM197106102842305 4930601

[B26] Lee FS Percy MJ The HIF pathway and erythrocytosis Annu Rev Pathol 2011 6 165 192 10.1146/annurev-pathol-011110-130321 20939709

[B27] McMullin MF HIF pathway mutations and erythrocytosis Expert Rev Hematol 2010 3 1 93 101 10.1586/ehm.09.68 21082936

[B28] Venegoni P Schroth G Myocardial infarction and polycythemia vera: how should we treat it? Cathet Cardiovasc Diagn 1994 32 3 259 261 10.1002/ccd.1810320313 7954775

[B29] Nand S Orfei E Pulmonary hypertension in polycythemia vera Am J Hematol 1994 47 3 242 244 10.1002/ajh.2830470320 7942794

[B30] Hornbein TF Long term effects of high altitude on brain function Int J Sports Med 1992 13 Suppl 1 S43 S45 1483787 10.1055/s-2007-1024589

[B31] Ohno H Kobayashi T Masuyama S Nakashima M Ohno H, Kobayashi T, Masuyama S, Nakashima M First International Consensus Group Meeting on Chronic Mountain Sickness (CMS) in Matsumoto Progress in Mountain Medicine and High Altitude Physiology 1998 Matsumoto, Japan: Press Committee of the 3rd World Congress on Mountain Medicine and High Altitude Physiology 166

[B32] Viscor G Ricar A Leal C Viscor G, Ricar A, Leal C International Working Group for Chronic Mountain Sickness Height: Proceedings of the 5th World Congress on Mountain Medicine and High Altitude Physiology 2003 Barcelona, Spain: Universitat de Barcelona 39 42

